# Flow Cytometry-Based Determination of Ploidy from Dried Leaf Specimens in Genomically Complex Collections of the Tropical Forage Grass *Urochloa* s. l.

**DOI:** 10.3390/genes12070957

**Published:** 2021-06-23

**Authors:** Paulina Tomaszewska, Till K. Pellny, Luis M. Hernández, Rowan A. C. Mitchell, Valheria Castiblanco, José J. de Vega, Trude Schwarzacher, Pat (J.S.) Heslop-Harrison

**Affiliations:** 1 Department of Genetics and Genome Biology, University of Leicester, University Road, Leicester LE1 7RH, UK; ts32@leicester.ac.uk (T.S.); phh4@leicester.ac.uk (P.H.-H.); 2 Rothamsted Research, Harpenden, Hertfordshire AL5 2JQ, UK; till.pellny@gmail.com (T.K.P.); rowan.mitchell@rothamsted.ac.uk (R.A.C.M.); 3 International Center for Tropical Agriculture (CIAT), Cali 6713, Colombia; l.hernandez@cgiar.org (L.M.H.); V.Castiblanco@cgiar.org (V.C.); 4 Earlham Institute, Norwich Research Park, Norwich NR4 7UZ, UK; Jose.De-Vega@earlham.ac.uk

**Keywords:** ploidy, flow cytometry, apomixis, dried specimens, *Brachiaria*, *Panicum*, tropical forage grasses

## Abstract

*Urochloa* (including *Brachiaria*, *Megathyrus* and some *Panicum*) tropical grasses are native to Africa and are now, after selection and breeding, planted worldwide, particularly in South America, as important forages with huge potential for further sustainable improvement and conservation of grasslands. We aimed to develop an optimized approach to determine ploidy of germplasm collection of this tropical forage grass group using dried leaf material, including approaches to collect, dry and preserve plant samples for flow cytometry analysis. Our methods enable robust identification of ploidy levels (coefficient of variation of G0/G1 peaks, CV, typically <5%). Ploidy of some 348 forage grass accessions (ploidy range from 2*x* to 9*x*), from international genetic resource collections, showing variation in basic chromosome numbers and reproduction modes (apomixis and sexual), were determined using our defined standard protocol. Two major *Urochloa* agamic complexes are used in the current breeding programs at CIAT and EMBRAPA: the ’*brizantha*’ and ’*humidicola*’ agamic complexes are variable, with multiple ploidy levels. Some *U. brizantha* accessions have odd level of ploidy (5*x*), and the relative differences in fluorescence values of the peak positions between adjacent cytotypes is reduced, thus more precise examination of this species is required. Ploidy measurement of *U. humidicola* revealed aneuploidy.

## 1. Introduction

Understanding the genome compositions of species within complexes including diploid and polyploid species is critical to evaluate their biodiversity, for conservation and to evaluate the potential for use in breeding; measurement of genome size, across potentially large germplasm collections, underpins such work. Grasslands and rangelands with grasses as the dominant species, being the largest ecosystems in the world, are the basic feed resources for livestock, and contribute to the livelihoods of over 800 million people including smallholders (Food and Agriculture Organization of the United Nations; http://www.fao.org/, accessed on 25 March 2021). Only 100–150 of the 10,000 forage species have been extensively cultivated, but many more have great potential for sustainable agriculture, and improvement and conservation of grasslands, including the genus *Urochloa* (previously classified in *Brachiaria*, and some *Eriochloa*, *Panicum* and *Megathyrsus*) [1] comprising species native to tropical and subtropical regions of Africa. The great forage potential of these grasses have been recognized in the 1950s [2], leading to the acquisition of 700 accessions of *Urochloa* and related genera during the joint collection mission of CGIAR (Consultative Group on International Agricultural Research) lead centers: CIAT (Centro Internacional de Agricultura Tropical) and ILRI (International Livestock Research Institute) in Africa in the 1980s. Five species of *Urochloa*: *U. ruziziensis*, *U. decumbens* and *U. brizantha* (belonging to the ‘*brizantha*’ agamic complex), *U. humidicola* (belonging to the ‘*humidicola*’ agamic complex), and *U. maxima* were then introduced in South America, and have been used as fodder plants mainly in Colombia and Brazil [3].

In exploiting biodiversity in breeding, improvements in yield and nutritional quality of forages can be achieved by identifying genes increasing the digestibility of plant cell walls and the protein and lipid content in vegetative tissues, and increasing biomass production [4]. By introduction to plant breeding programmes, genetic improvement of forage lines, recurrent genetic selection of plants showing useful traits, and subsequent hybridizations and back-crossings [5,6], create more diverse agroecosystems resilient to climate and environmental changes [7]. The DNA amount measurement for ploidy and genome size estimation, and the characterization of genome composition are required for effective use of diploids and polyploids in breeding programs, as well as for research purposes [8,9].

Preparation of metaphases from dividing plant tissues, followed by microscopy and chromosome counting, is widely used to determine the ploidy of individual plants and show polyploid series within larger groups. However, the method is time-consuming and highly skilled, both in terms of growing plants and collecting root-tips or meiotic material, and in making the preparations. The most rapid and convenient technique for ploidy measurement is flow cytometry using suspensions of fluorescently labeled nuclei [10,11,12,13,14], that is now widely adopted for fresh leaf specimens.

Phenolics, hydroxamic acids, and short-chain fatty acids are present in plants, and some of these phytochemicals have been identified as inhibitors of fluorescent DNA staining, hence leading to inaccurate flow cytometry-based measurement of DNA content [15,16,17,18,19]. The ability of tropical and subtropical plants to synthesize secondary metabolites and possess allelopathic potential is exceptional [20]. Seasonal and regional differences in accumulation of secondary products may cause differences in staining for flow cytometry. Secondary metabolites and their phytotoxicity on forage legumes have been recognized in *Urochloa* tropical forage grasses [21,22,23,24], which has been suggested to make it difficult to analyze these plants by flow cytometry [25].

For *Urochloa*, ploidy estimation across the whole germplasm collection (excluding one species, *U. ruziziensis*, known only as a diploid) is required due to the different pathways of reproduction showing sexual and apomictic accessions within same species [26], natural triploid interspecific hybrids [27], different genome compositions both within and between species [9], confirmed aneuploidy [9,28,29], and different basic chromosome numbers (*x* = 6, 8 and 9) [30,31,32,33,34,35].

Ideally, a common reference standard for flow cytometry and ploidy measurement should be a diploid plant from the taxon of the tested samples, grown and collected under similar conditions, and where chromosomes can be prepared and counted. For a pool with diverse ploidies, several standards are helpful, although the lack of seeds or living plants may make it impossible to prepare metaphase plates from root tips, and challenges (e.g., due to apomictic mode of reproduction of studied species, difficulties with germination of tropical plant seeds, or having only herbarium samples), may mean a less related standard with known ploidy level, genome size and basic chromosome number similar to the unknown samples must be used [14].

Fresh leaves have usually been considered the best material for flow cytometry analysis. However, there is often a requirement for use of field-material, collected under suboptimal demanding conditions compared to plants for greenhouse or experimental field and requiring storage and transport to the flow cytometry facility. Additionally, work often needs to use herbarium or stored material, which may not be possible to collect again, or is the reference for published studies, or is determined as a new species/taxonomic revision, requiring determination of ploidy and estimation of genome size [36]. The applicability of flow cytometry for dehydrated leaves is limited by several factors, including insufficient amounts of tissue, sampling of mature plants, incorrect drying, storage and preservation of samples, and the low efficiency of nuclei isolation due to their degradation.

For flow cytometry analysis of nuclear genome sizes from fresh and dried material, coefficient of variation of the G0/G1 peak (CV), that quantifies a precision of individual flow cytometry measurements, is an important criterion showing estimation of nuclei integrity and variation in DNA staining [37,38,39,40,41,42]. Low coefficient of variation, even for dried leaf specimens [36], or seeds [19], petals and pollen [43], can be achieved by using appropriate isolation buffers and their supplementation, stains and staining protocols, the practical technique used for chopping leaves in the buffer, and even choice of razor blades. 

Here, we aimed to develop an optimized and robust approach to determine ploidy of tropical forage grasses using dried leaf material. The established method can be widely adopted for dried leaf specimens, especially when screening genomically variable germplasm resource collections, defining a standard protocol recommendation. More specifically, we intended to optimize the flow cytometry assay for *Urochloa* grass group that shows variation in basic chromosome numbers and reproduction modes.

## 2. Materials and Methods

### 2.1. Plant Material

**Figure 1:**
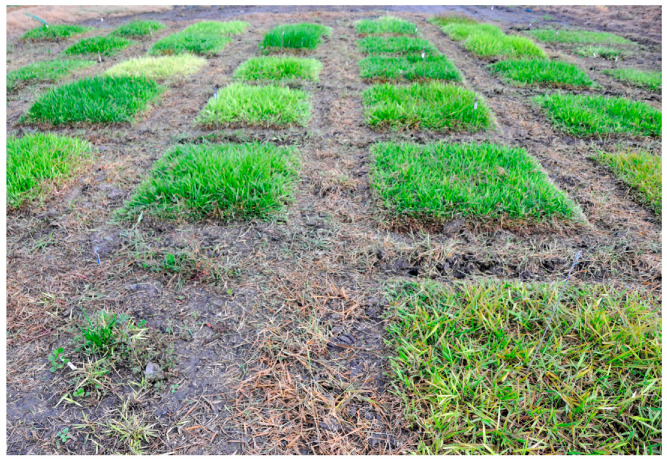
Field plots of *Urochloa* tropical forage grasses in CIAT, Colombia.

Accessions of *Urochloa* and related species used in the study are listed in Supplementary Data Table S1. Plants used as external standards, and their seed providers are given in Table 1. Leaf samples intended for flow cytometry analysis were collected from germplasm accessions grown and maintained in the field genebank at CIAT Palmira campus (Figure 1).

### 2.2. Collection and Preservation of Plant Material for Flow Cytometry

1. Leaf fragments of approximately 1 g fresh weight were harvested in the field, folded into permeable manila seed storage envelopes (80 gsm) and kept in a sealed plastic bag on wet ice. Young leaves from typical vigorous specimens, representative of the population in each plot, were selected. Insect damaged and discolored plants were avoided.
2. The envelopes were then stored in a sealed desiccator at ambient pressure, or an airtight plastic box (as used for sandwiches or larger sizes), at room temperature with a thick layer of self-indicating silica gel (a granular material with c. 3–5 mm irregular beads; Type III Sigma-Aldrich, S7625; or self-indicating mixed with non-indicating silica gel, cheaply available from online marketplaces). The silica gel was changed daily until it did not change color, which was after approximately 4–5 days. In total, 250 g of silica gel was used for 30 leaf samples.
3. Multiple samples in the paper envelopes were then transferred to sealed plastic bags with a small amount of silica gel.
4. The plastic bags with envelopes of dried leaves and silica gel were shipped under ambient conditions to the University of Leicester, UK (with appropriate export and import documentation, here under “Section IV: Cut flowers, foliage and vegetables” and “Section III: Seeds for planting” of the UK “Import requirements for plants, plant produce and products”). The sealed bags, after inspection and replacement of silica gel if required, were then stored in 4 °C in plastic boxes containing silica gel until flow cytometry analysis.
5. The seeds received from VIR, USDA and CIAT were germinated in a tropical greenhouse (25 °C), and leaf samples were collected from plants, and dried and preserved in the same way as those collected in the field in Colombia, and then used as standards for flow cytometry analysis.

### 2.3. Sample Preparation for Flow Cytometry

Cell nuclei suspensions from dehydrated leaf tissues were prepared for flow cytometric analysis according to Doležel et al. [44] with minor modifications:

1. In total, 500 mg of dried leaf of each accession were chopped with a sharp razor blade in a 55 × 15 mm polystyrene Petri dish with ice cold 1 mL nucleus-isolation buffer. Much smaller amounts of leaf material (100 mg) did not give suitable nuclear suspensions. We used double edge stainless razor blades, allocating one razor edge per one studied accession. For safe holding of the razor blade while chopping, a rubber grip was used.
2. Three different standard buffers were evaluated, as shown in Table 2. Buffers were supplemented with 15 mM β-mercaptoethanol (Calbiochem CAS 60-24-2) and 1% PVP-40 (polyvinylpyrrolidone-40; Sigma PVP40) and the effect of these chemicals on reducing the negative effect of cytosolic and phenolic compounds was tested.
3. After finely chopping the material in the buffer, the nuclei suspension was passed through a 50 μm mesh nylon filter (CellTrics, Sysmex 04-004-2327) into the 12 × 75 mm round-bottom polystyrene flow cytometry tubes (Falcon^®^ 352052, with caps preventing cross-contamination), and placed on ice.
4. The nuclei suspension was then supplemented with propidium iodide (PI, Sigma P4170; 50 µg mL^−1^; solution in deionized water, passed through a 0.22 mm filter), and ribonuclease A (Sigma R6513; 50 µg mL^−1^) to prevent staining of double-stranded RNA, and mixed gently using vortex.
5. Samples were incubated for at least 10 min on ice in darkness, and then were analyzed in an Accuri C6 Flow Cytometer (Becton Dickinson), equipped with a 20 mW laser illumination operating at 488 nm.

### 2.4. Flow Cytometry Analysis

**Figure 2:**
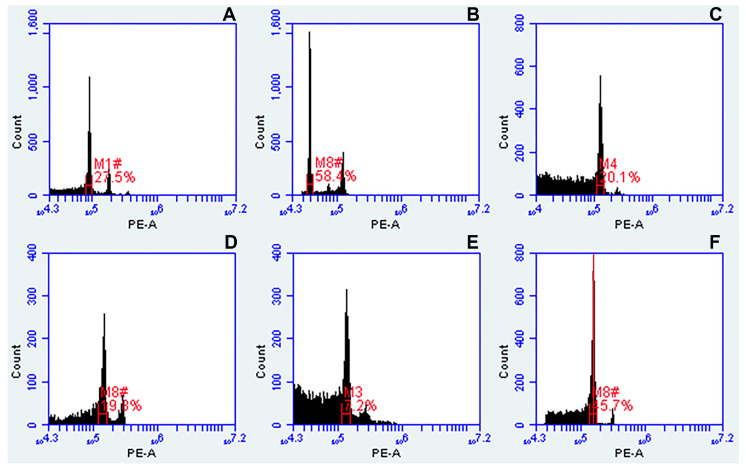
Optimization of flow cytometry assay for dried leaf samples. (**A**) Three gradually declined peaks of diploid *U. decumbens* CIAT 26185 indicating endoreduplication; (**B**) additional high peak in histogram of tetraploid *U. maxima* CIAT 16055, indicating contamination of leaf sample; (**C**) fresh and (**D**) dried leaf samples of *Panicum miliaceum* showing the same position of peaks in histograms, but slight differences in number of nuclei and CV of DNA peaks; (**E**) histogram of dried leaf sample of *Panicum miliaceum* with no gating tools applied; (**F**) histogram of dried leaf sample of *Panicum miliaceum* where gating tools were applied, giving sharp peaks and low background. Here, gain or peak height was adjusted using a software function (VirtualGain) and the center of the peak selected as reference point (red line; Peak Definition Marker). Regions of identification (red marks) were placed across the peaks to export fluorescence values representing peak positions and CVs (coefficient of variation of G0/G1 peaks).

In the first stage of the analysis, it was checked whether the dry and fresh samples gave the same results (fluorescence value of peak positions). The dry leaves were then used for the daily calibration of flow cytometer. For ploidy measurement, *Panicum miliaceum* Mil69 (2*n* = 4*x* = 36) [45] was used as a primary external standard to recognize ploidy of some accessions of studied plants. Subsequent external standards were then included in the analyses, and their number of chromosomes was confirmed microscopically. We used the following protocol for the ploidy measurement:

1. Prior to measuring the ploidy of samples from a given species, the flow cytometer was calibrated using the specified external standard (Table 1). For calibration, we used VirtualGain tool. In the external standard plot, the Peak Definition Marker (see red line in Figure 2F) was manually moved to the center of the external standard peak, becoming the reference point. Samples to be measured were aligned to this position.
2. The histograms (FSC-A vs. SSC-A, FL1-A vs. FL2-A, FL3-A vs. FL2-A, and a univariate histogram of FL2-A) were acquired using the CFlow^®^ Plus software set up according to Galbraith and Lambert [46]. Here, the following filter configurations were used: FL-1-a 530/14-nm bandpass filter; FL-2-a 585/20-nm bandpass filter; FL-3-a 670-nm longpass filter. The primary threshold was set to channel 10,000 on FSC-A to gate out debris and noise from nuclei suspension. The secondary threshold was set at 1000 for FL-2. Polygonal gating tool was used to draw a region on the FSC-A vs. SSC-A plot, and a line-shaped cluster of dots showing PI-stained nuclei in the biparametric dot plot of FL2-A vs. FL3-A. Based on this gating, G0/G1 and G2 peaks appeared in a univariate histogram of FL2-A.
3. The relative fluorescence values of the peak positions of PI-stained nuclei (FL) and the coefficient of variation (CV) of the G0/G1 peak to estimate nuclei integrity and variation in DNA staining were evaluated in each sample by manually placing regions of identification across the peak to export values.
4. Ploidy of studied plants was determined by comparing the fluorescence values of the peak positions of samples to that of external standards.

### 2.5. Microscopy and Validation of Chromosome Numbers

For chromosome number calculation of external standards, we used a modified protocol of Schwarzacher and Heslop-Harrison [50]:

1. *Urochloa* seeds, like many other tropical grasses, did not germinate in Petri dishes. The seeds were germinated in a 25 °C greenhouse, in 15 × 15 cm plastic pots containing Levington F2+S soil.
2. Root tips were collected from plants cultivated in a greenhouse, treated with α-bromonaphthalene (Sigma B73104) at room temperature for 2 h, and 4 °C for 4 h, and fixed in absolute ethyl alcohol:acetic acid solution, 3:1.
3. The root tips were washed in enzyme buffer (10 mM citric acid/sodium citrate) for 15 min, and then they underwent enzymatic maceration in 20 U/mL cellulase (Sigma C1184), 10 U/mL ‘Onozuka’ RS cellulase (RPI C32400) and 20 U/mL pectinase (Sigma P4716 from *Aspergillus niger*; solution in 40% glycerol) in 10 mM enzyme buffer for 60 min at 37 °C.
4. Digested root tips were squashed in 60% acetic acid. Cover slips were removed after freezing with dry ice.
5. Air-dried slides were counterstained with DAPI (4′,6-diamidino-2-phenylindole, Sigma D9542; 2 µg mL) in antifade solution (Citifluor, Vectashield, Slowfade or any other commercial antifading reagents for fluorescence microscopy), which prevents the permanent loss of fluorescence due to prolonged exposure to high intensity light sources.
6. Slides were analyzed with an epifluorescence microscope with appropriate UV illumination, filters and camera (Nikon Eclipse 80i; DS-QiMc monochromatic camera, and NIS-Elements v.2.34 software, Nikon, Tokyo, Japan). The number of chromosomes was counted for approximately 50 metaphases derived from 5 plants of each accession used as potential external standard for flow cytometry.

## 3. Results

### 3.1. Optimization of Flow Cytometry Assay for Dried Leaves of Urochloa

#### 3.1.1. Flow Cytometry Troubleshooting

Nuclei isolated from properly collected, dried and well-preserved leaf samples, as explained in materials and methods, gave histograms showing peaks from cells at different stages of the cell cycle: higher G0/G1 (2C) and lower G2 (4C) peaks. Sometimes more peaks were observed, like three gradually declined peaks in Figure 2A, indicating endoreduplication process. Use of older leaf collections gave DNA peaks having very high CVs (coefficient of variation of G0/G1 peaks), above 10%, or additional peaks on the histogram (Figure 2B), and these samples were excluded from further analysis. Fresh and dried leaves gave very similar position of peaks, as shown in Figure 2C,D for comparison, however, sometimes the number of isolated nuclei from dried leaves was slightly smaller due to sample degradation. Several samples of one accession, where possible, were run as the position of the peak in the histogram may vary slightly between plants (Supplementary Data Table S1). In the example of Figure 2F, nuclei of interest were being selected (gated) in the FSC-A vs. SSC-A and FL2-A vs. FL3-A plots (as explained in materials and methods), resulting in sharper peaks of G0/G1 and G2 and lower background in univariate histogram of FL2-A, in comparison to Figure 2E where gating tools were not applied.

#### 3.1.2. Buffers

**Figure 3:**
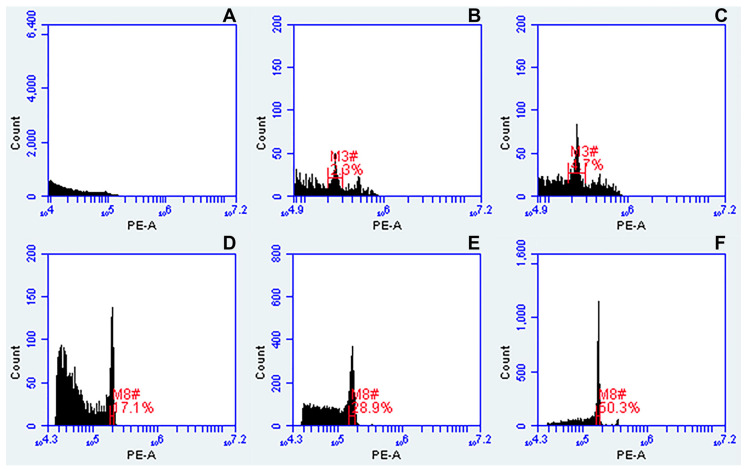
Comparison of three different standard buffers for nuclei isolation from dried leaves of tetraploid *Urochloa* accessions, and their effect on histogram quality. (**A**) Galbraith’s buffer; (**B**) Otto’s buffer; (**C**) Otto’s buffer supplemented with 15 mM β-mercaptoethanol and 1% PVP-40 (Modified Otto, see Table 2); (**D**) Partec buffer; (**E**) Partec buffer supplemented with β-mercaptoethanol; (**F**) Partec buffer supplemented with 15 mM β-mercaptoethanol and 1% PVP-40 (Modified Partec, see Table 2). Regions of identification (red marks) were placed across the peaks to export fluorescence values representing peak positions and CVs.

Three different standard isolation buffers (Table 2) were tested to isolate nuclei from dehydrated *Urochloa* leaves. No peaks (Figure 3A) or very low peaks were obtained analyzing samples of nuclei isolated using Galbraith’s buffer [47] which is optimized for fresh material. Small numbers of nuclei were isolated using Otto’s buffer [48] giving histograms with increased level of background and high CVs (Figure 3B). Supplementation of Otto’s buffer with 15 mM β-mercaptoethanol and 1% PVP-40 only slightly increased the peak resolution (Figure 3C). Well-defined histograms with acceptable CV values and reasonable number of nuclei (Figure 3D–F) were obtained using Partec buffer [49]. The sharpest peaks were yielded after supplementation of this buffer with 15 mM β-mercaptoethanol and 1% PVP-40 (Figure 3F).

#### 3.1.3. External Standards Used for Flow Cytometry Analysis

**Figure 4:**
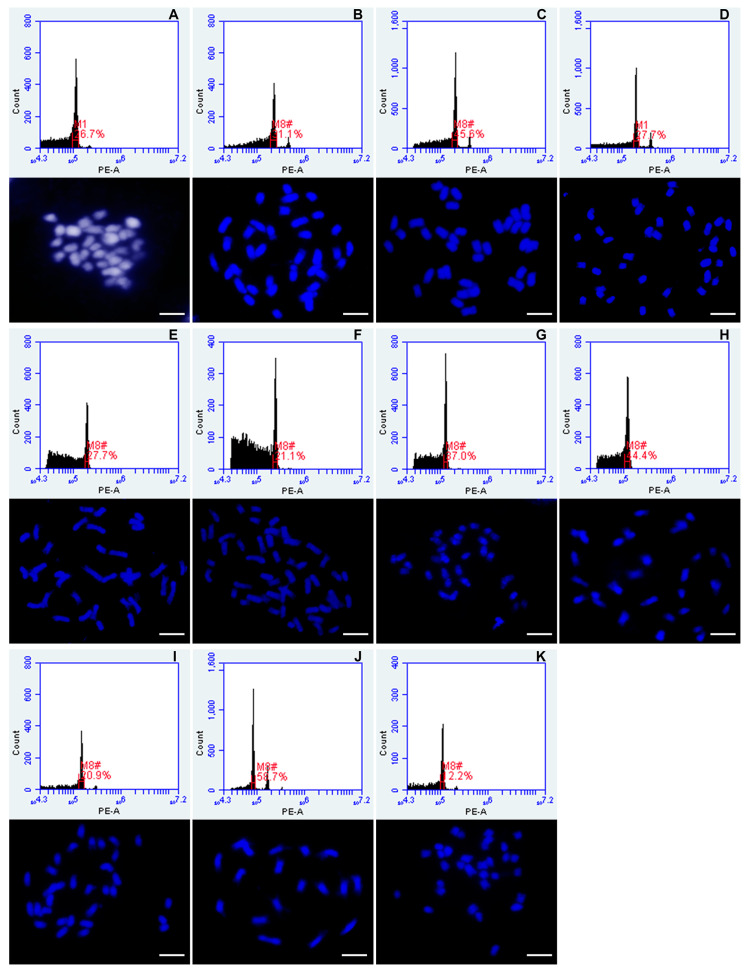
Histograms of relative fluorescence intensities showing ploidy levels and the corresponding chromosome numbers of different genotypes selected as potential external standards for flow cytometry analysis of *Urochloa* germplasm collection. (**A**) *Panicum miliaceum* Mil69 (2*n* = 4*x* = 36); (**B**) *Urochloa brizantha* PI 292187 (2*n* = 4*x* = 36); (**C**) *Urochloa decumbens* CIAT 664 (2*n* = 4*x* = 36); (**D**) *Urochloa decumbens* CIAT 6370 (2*n* = 4*x* = 36); (**E**) *Urochloa humidicola* CIAT 26151 (2*n* = 6*x* = 36); (**F**) *Urochloa humidicola* CIAT 16867 (2*n* = 8*x* + 2 or 9*x* − 4 = 50), recognized as aneuploid and thus cannot be used as external standard; (**G**) *Urochloa maxima* CIAT 6171 (2*n* = 4*x* = 32); (**H**) *Urochloa maxima* CIAT 16004 (2*n* = 4*x* = 32); (**I**) *Urochloa maxima* PI 284156 (2*n* = 4*x* = 32); (**J**) *Urochloa ruziziensis* CIAT 6419 (2*n* = 2*x* = 18); (**K**) *Urochloa* sp. PI 657653 (2*n* = 4*x* = 32). Regions of identification seen in plots (red marks) were placed across the peaks to export fluorescence values representing peak positions and CVs. Scale bars = 5 µm.

The procedure of ploidy determination of *Urochloa* species using flow cytometry of dried leaf specimens was optimized by choosing appropriate buffer composition (Table 2, Figure 3), drying and preservation of plant samples (protocol in material and methods, Figure 2), chopping technique (Figure 2) and different external standards (Table 1). *Panicum miliaceum* Mil69 (2*n* = 4*x* = 36) was used as a primary external standard to recognize accessions for which the level of ploidy was certain. Ten different accessions were selected as potential external standards. The ploidy of these plants was validated by preparing mitotic slides and counting chromosomes microscopically (Figure 4). The position of peaks of *Urochloa humidicola* CIAT 16867 in the histogram (Figure 4F) suggested that this accession was most likely to be heptaploid, but chromosome counting revealed it to be aneuploid with 2*n* = 8*x* + 2 or 9*x* − 4 = 50, thus, this accession was excluded from further analysis. The other nine accessions were then used as external standards, and their mean fluorescence values of the peak positions are given in Table 1.

### 3.2. Ploidy Measurement of Urochloa Species

**Figure 5:**
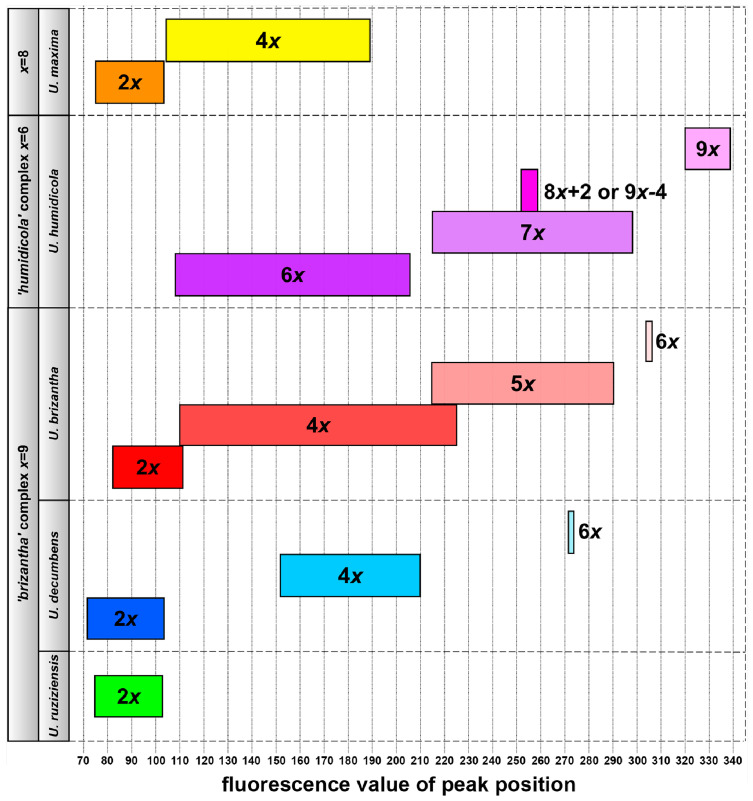
Ranges of fluorescence values of the peak positions for different ploidy levels of the most numerous species (*‘brizantha’* agamic complex: *U. ruziziensis, U. decumbens, U. brizantha*; ’*humidicola*’ agamic complex: *U. humidicola*; *U. maxima*) in CIAT germplasm collection.

DNA content of 348 accessions of different *Urochloa* species were measured using flow cytometry of imported dried leaf materials using the optimized technique giving very sharp peaks. Fluorescence values of the peak positions and CVs were exported and are given in Supplementary Data Table S1 and summarized in Table 3. CV values were slightly increased comparing to the fresh leaf specimens of *Panicum miliaceum* (CV 2.5%). Where possible, several leaf samples for one accession were measured enabling comparison of fluorescence values of the peak positions between different plants of the same accession. In general, these values did not differ significantly from plant to plant, proving the established method. The fluorescence values of the peak positions of samples were compared to that of the nine standards (Table 1), which allowed to establish that other species with a similar genome size and basic chromosome numbers can be used as external standards for ploidy measurements of our studied accessions. For each level of ploidy of the individual species, a range of fluorescence values of the peak positions were established (Table 3), and these ranges for the most numerous species are shown in Figure 5.

#### 3.2.1. ‘Brizantha’ Agamic Complex

Three species belonging to the ‘*brizantha*’ agamic complex, *Urochloa ruziziensis*, *U. decumbens* and *U. brizantha*, have a basic chromosome number *x* = 9. All accessions of *U. ruziziensis* studied here were diploid (Supplementary Data Table S1), showing similar range of fluorescence values of the peak positions to that of diploid *U. decumbens* (see Table 3 and Figure 5). Within both species there are single samples showing higher fluorescence values of the peak positions than the others. *U. decumbens* accessions differ in their ploidy levels, showing diploids, tetraploids, and hexaploid (Supplementary Data Table S1), that can be clearly distinguishable using flow cytometry, because the ranges of fluorescence values of the peak positions for each ploidy level did not overlap. This result contrasts with *U. brizantha*, where the ranges the fluorescence values of the peak positions of diploids, tetraploids, pentaploids and hexaploids overlapped, meaning that ploidy levels of this species are not so obvious (see Figure 5). This is particularly evident when looking at the differences in fluorescence values between samples of the same accession (see Supplementary Data Table S1). 

#### 3.2.2. ‘*Humidicola*’ Agamic Complex

Two polyploid species with basic chromosome number *x* = 6 were assigned to the ‘humidicola’ agamic complex: U. humidicola and U. dictyoneura. Three different ploidy levels were recognized in the U. humidicola: hexaploid, heptaploid, and nonaploid (Supplementary Data Table S1). U. dictyoneura accession used in our studies seemed to be heptaploid. In general, each ploidy level of U. humidicola has its own range of fluorescence values of the peak positions (Figure 5), however, due to confirmed aneuploidy within species (U. humidicola CIAT 16867 with 2*n* = 8*x* + 2 or 9*x* − 4 = 50), additional validation, e.g., counting chromosome numbers, would be needed.

#### 3.2.3. Urochloa Maxima

Two ploidy levels were recognized in *U. maxima*. Some diploid and tetraploid accessions showed similar fluorescence values of the peak positions (Figure 5). Those samples that have extreme results and peaks well beyond those of the reference external standards should have their chromosomes counted.

#### 3.2.4. Related Species

Several tropical grass species with potential for improvement and wider use as forages were studied here, including other cultivated and wild *Urochloa* species: *U. arrecta, U. dura, U. jubata, U. nigropedata, U. plantaginea*, and *U. platynota*. In our analysis, we also used one *Urochloa* accession not assigned to species (possible hybrid) and one synthetic multigeneration hybrid involving *U. ruziziensis*, *U. decumbens* and *U. brizantha*, both accessions having huge potential for sustainable grazing and pasture management. In all cases, our external standards were useful to establish ploidy levels of studied species. 

## 4. Discussion

### 4.1. Flow Cytometry as a Useful Tool for Measuring the Ploidy of Large Germplasm Pool

Flow cytometry has become the standard technology for measuring the ploidy and genome sizes of plants [40], allowing the measurement of hundreds of samples, even genomically diverse species, in a relatively short time. In most cases, freshly collected, field- or garden-grown leaf material is used, with a small number of accessions. We optimized the methods for sampling, drying, storage, transport and preservation of tropical forage grasses to use some time later with a robust flow cytometry protocol for measurement of ploidy. We show the utility in a relatively large and diverse germplasm collection (348 accessions) of the tropical forage grass genus *Urochloa* (previously placed in genus *Brachiaria* and some *Eriochloa* and *Panicum*) [51,52]. Our analysis proved that the requirement of fresh leaf material is not a crucial limitation for flow cytometry-based determination of ploidy, especially while screening a large germplasm pool [36,43,53]. The method allows wider field and geographical sampling of plants when fresh leaf tissues cannot be examined shortly after harvesting [54]. 

### 4.2. Needs for Screening Urochloa Germplasm Collection

Integration of ploidy levels and agronomic traits, especially those related to resistance and tolerance to pest and diseases, is important to define a breeding strategy to exploit germplasm with diverse ploidy levels [5,55,56]. Where collections have various ploidies, flow cytometry can help the verification of samples from field collections, where mislabeling, or spread of incorrect seed or plants in vegetative plots may lead to replacement of one accession with another over decades. Comparison of similar accessions numbers from Brazil and Colombia detects some such differences. Polyploidy promotes genome diversification and gives plasticity to species [57], thus it is pertinent to examine ploidy of as many accessions as possible in order to choose those suitable for crossbreeding. For research purposes, sampling and screening the large germplasm collections provides additional characters and helps to better estimate genome relationships between species within large plant complexes, such as *Urochloa* [1] and hence help reconstruct phylogenies, particularly those where reticulate evolution of polyploid taxa is found. Flow cytometry and the measurement of nuclear DNA contents has other applications not considered here, in particular for analysis of the cell cycle [58], and examining differentiation of cells through endopolyploidy [59]. 

### 4.3. Choice of Flow Cytometry Approaches to Determine Ploidy from Dried Leaf Specimens

We developed an optimized approach to determine ploidy of germplasm collection of the tropical forage grass genus *Urochloa* using dried leaf material. The analysis showed that our method enabled robust identification of ploidy levels from dehydrated specimens. The coefficient of variation of G0/G1 peaks (CV) of the samples studied here, was typically <5%, which is desirable, but analysis of much older leaves, e.g., herbarium specimens, can give broader peaks with a still usable CV between 5% and 10% [44]. 

The most suitable buffer for estimation of ploidy levels from both fresh and dried *Urochloa* leaves was Partec. Its components include Tris as buffer, NaCl (85 mM) to maintain nuclear integrity, β-mercaptoethanol as antioxidant, and PVP-40 (polyvinylpyrrolidone-40) to bind polyphenols and anthocyanins, scavenge other polar molecules and deactivate proteins from the plant cells; Triton X-100 as a detergent to aid buffer penetration. The Partec buffer was successfully used on plants rich in secondary metabolites, such as *Lupinus*, *Brassica*, *Cocos, Matricaria* or *Cyclopia*. In other species, however, this buffer did not produce peaks, and the high concentration of specific polyphenols in *Aspalathus* inhibited nuclei isolation [60], thus each group of plants requires several buffers to be tested to obtain very sharp peaks and reliable results. 

In our ploidy analysis we used propidium iodide (PI) which is one of the most widely used fluorescence reagents in flow cytometry binding to DNA by intercalating between DNA bases and showing no AT or GC preference. Its fluorescence with green-light excitation is enhanced some 20-fold when bound to DNA compared to in solution. The emission maximum depends on the solvent, and in the aqueous solution used for nuclear isolation, the maximum is 636 nm (red) [61]. PI shows intermolecular proton transfer reaction in solvent; it interacts with SDS (sodium dodecyl sulphate), thus we avoided this widely used detergent and did not use it as a component of a nuclei isolation buffer for flow cytometry. Propidium iodide also binds to RNA, showing enhanced fluorescence (with a slightly different fluorescent color), so for nuclear staining, RNase needs to be added to the buffer. In practice, the concentration of PI and RNase in the buffer is important, and peaks broaden (higher CV) when they are too high or too low. In our analysis we used exactly the same concentration of PI and RNase, which allowed to obtain reliable results.

### 4.4. Urochloa Germplasm Findings

Here, we verified ploidy of 348 accessions of different *Urochloa* species, which represent a significant proportion of CIAT germplasm resources. However, determining the ploidy levels of grass group showing both apomictic and sexual mode of reproduction, like *Urochloa*, can become a challenge and requires the use of appropriate standards of known ploidy and number of chromosomes [62]. For *Urochloa* grass complex, different internal standards were needed due to the different genome sizes within and between agamic complexes and species, and different basic chromosome numbers (Supplementary Data Table S1). The average DNA content and genome sizes given as Cx values have been published already for *Urochloa* species [27,63]. Most diploid accessions of *U. brizantha* studied here are apomict [9], showing larger fluorescence values of the peak positions than sexual diploid accessions of *U. decumbens* and *U. ruziziensis*, proving that the genome size depends on the mode of reproduction [63], which is an additional challenge for screening diverse germplasm collections. While in diploid and polyploid accessions of *U. decumbens* a small shift in peak position on histogram usually does not compromise reliability of ploidy estimates, attention should be paid to the analysis of *U. brizantha* showing odd ploidy levels, because relative differences in fluorescence values of the peak positions between neighboring cytotypes (2*x*, 4*x*, 5*x*, 6*x*) is decreased; such a phenomenon is also observed in species with ploidy levels greater than 6x [44]. A more precise examination of *U. humidicola* is also required due to confirmed aneuploidy [28,29], odd ploidy levels [34], and unrecognized diploid ancestors [64].

## 5. Conclusions

*Urochloa* tropical forage grasses have a great potential for sustainable agriculture and intensive grazing management of cover crops. Some of them are included in the current breeding programs at CIAT and EMBRAPA, now mainly focused on crossing tetraploids within ‘*brizantha*’ and ‘*humidicola*’ agamic complexes and *Urochloa maxima* [65], but other species studied here can also be included and used as fodder plants. This tropical forage grass group is genomically complex [9], having species recognized as being very variable in number of chromosomes, and ploidy levels which is the result of apomictic reproduction, and reflecting the genetic diversity present in a given population [66]. The ploidy levels of some *Urochloa* accessions have been previously measured [25,65,67,68,69], but some data vary between papers and reports [9]: thus, values may require checking for a particular accession name.

## Figures and Tables

**Table 1:** External standards used for flow cytometry analysis of *Urochloa* germplasm collection. Chromosome numbers were counted microscopically.

External Standard	Number of Chromosomes	Mean Fluorescence Values of the Peak Positions	Sample Species
*Panicum miliaceum* VIR, Mil69	2*n* = 4*x* = 36	112	-
*Urochloa brizantha,* USDA, PI 292187	2*n* = 4*x* = 36	225	*U. brizantha, U. dura, U. platynota, U. ruziziensis* × *U. decumbens* × *U. brizantha* hybrid
*Urochloa decumbens,* CIAT, 664	2*n* = 4*x* = 36	205	*U. arrecta, U. decumbens, U. jubata, U. nigropedata, U. plantaginea, U. ruziziensis*
*Urochloa decumbens,* CIAT, 6370	2*n* = 4*x* = 36	194	*U. arrecta, U. decumbens, U. jubata, U. nigropedata, U. plantaginea, U. ruziziensis*
*Urochloa humidicola,* CIAT, 26151	2*n* = 6*x* = 36	197	*U. humidicola, U. dictyoneura*
*Urochloa maxima,* CIAT, 6171	2*n* = 4*x* = 32	131	*U. maxima, U. nigropedata*
*Urochloa maxima,* CIAT, 16004	2*n* = 4*x* = 32	120	*U. maxima, U. nigropedata*
*Urochloa maxima,* USDA, PI 284156	2*n* = 4*x* = 32	149	*U. maxima, U. nigropedata*
*Urochloa ruziziensis,* CIAT, 6419	2*n* = 2*x* = 18	83	*U. arrecta, U. decumbens, U. jubata, U. nigropedata, U. plantaginea, U. ruziziensis*
*Urochloa* sp., USDA, PI 657653	2*n* = 4*x* = 32	111	*U. maxima*

United States Department of Agriculture (USDA, USA); Vavilov Research Institute (VIR, St Petersburg, Russia); Centro Internacional de Agricultura Tropical (CIAT, Colombia).

**Table 2:** Nuclei isolation buffers and their compositions.

Buffer	Composition
Galbraith [47]	45 mM MgCl_2_, 20 mM MOPS, 30 mM sodium citrate, 0.1% (*v*/*v*) Triton X-100 (pH 7)
Otto [48]	Otto I: 100 mM citric acid, 0.5 % (*v*/*v*) Tween 20 (pH 2–3)Otto II: 400 mM Na_2_PO_4_ · 12H_2_O (pH 8–9)
Modified Otto (this paper)	Otto I: 100 mM citric acid, 0.5 % (*v*/*v*) Tween 20 (pH 2–3)Otto II: 400 mM Na_2_PO_4_ · 12H_2_O (pH 8–9), 15 mM β-mercaptoethanol, 1% PVP-40
Partec [49]	100 mM Tris, 2.5 mM MgCl_2_ · 6H_2_O, 85 mM NaCl, 0.1% (*v*/*v*) Triton X-100 (pH 7,0)
Modified Partec (this paper)	100 mM Tris, 2.5 mM MgCl_2_ · 6H_2_O, 85 mM NaCl, 0.1% (*v*/*v*) Triton X-100 (pH 7.0), 15 mM β-mercaptoethanol, 1% PVP-40

**Table 3:** Variation in fluorescence values of the peak positions between species, for the different ploidy levels determined.

Species	Ploidy	Number of Studied Accessions	Number of Studied Plants	Range of Fluorescence Values of the Peak Positions	Mean Fluorescence Values of the Peak Positions	CV (%) Range	CV (%) Average
*Urochloa arrecta*	2*n* = 4*x*	1	1	93	93	7.53	7.53
*Urochloa brizantha*	2*n* = 2*x*	6	9	82–110	96	5.46–9.14	7.32
	2*n* = 4*x*	59	70	111–225	172	2.9–9.89	5.65
	2*n* = 5*x*	25	37	215–291	247	3.4–8.17	5.34
	2*n* = 6*x*	1	1	303	303	3.83	3.83
*Urochloa decumbens*	2*n* = 2*x*	18	19	72–104	87	2.68–6.87	4.76
	2*n* = 4*x*	25	28	152–211	183	3.25–5.66	4.52
	2*n* = 6*x*	1	1	270	270	4.02	4.02
*Urochloa dictyoneura*	2*n* = 7*x*	1	1	220	220	5.91	5.91
*Urochloa dura*	2*n* = 5*x*	1	2	255–282	268	4.82–5.18	5
*Urochloa humidicola*	2*n* = 6*x*	16	21	108–205	174	3.69–6.24	4.65
	2*n* = 7*x*	33	45	215–298	259	2.84–6.4	4.31
	2*n* = 8*x* + 2 or 9*x* − 4	1	2	253–259	256	3.04–3.49	3.27
	2*n* = 9*x*	3	4	320–338	330	3.39–5.33	4.7
*Urochloa jubata*	2*n* = 2*x*	1	1	87	87	5.89	5.89
	2*n* = 4*x*	1	1	123	123	4.73	4.73
*Urochloa maxima*	2*n* = 2*x*	25	31	74–104	94	4.81–9.23	7.02
	2*n* = 4*x*	99	102	104–190	128	3.73–8.81	5.5
*Urochloa nigropedata*	2*n* = 4*x*	1	2	142–146	144	3.91–6.53	5.22
*Urochloa plantaginea*	2*n* = 2*x*	1	1	90	90	6.62	6.62
*Urochloa platynota*	2*n* = 2*x*	1	1	98	98	5.96	5.96
*Urochloa ruziziensis*	2*n* = 2*x*	26	33	75–103	86	2.42–6.92	4.32
*Urochloa ruziziensis* × *Urochloa decumbens* × *Urochloa brizantha*	2*n* = 4*x*	1	1	190	190	2.67	2.67
*Urochloa* sp. PI657653	2*n* = 4*x*	1	1	111	111	4.42	4.42

## Data Availability

Not applicable.

## Supplementary Materials

The following are available online at https://www.mdpi.com/article/10.3390/genes12070957/s1, Supplementary Data Table S1. List of accessions used in the study, their fluorescence values of the peak positions, and coefficient of variations (CVs) of the G0/G1 peaks.
